# Face-Based Attention Recognition Model for Children with Autism Spectrum Disorder

**DOI:** 10.1007/s41666-021-00101-y

**Published:** 2021-07-15

**Authors:** Bilikis Banire, Dena Al Thani, Marwa Qaraqe, Bilal Mansoor

**Affiliations:** 1 Division of Information and Computing Technology, College of Science and Engineering, Hamad Bin Khalifa University, Qatar Foundation, Doha, Qatar; 2 Mechanical Engineering Program, Texas A & M University at Doha, Qatar, Doha, Qatar

**Keywords:** Facial landmarks, Geometric features, Attention recognition, ASD, Machine learning

## Abstract

Attention recognition plays a vital role in providing learning support for children with autism spectrum disorders (ASD). The unobtrusiveness of face-tracking techniques makes it possible to build automatic systems to detect and classify attentional behaviors. However, constructing such systems is a challenging task due to the complexity of attentional behavior in ASD. This paper proposes a face-based attention recognition model using two methods. The first is based on geometric feature transformation using a support vector machine (SVM) classifier, and the second is based on the transformation of time-domain spatial features to 2D spatial images using a convolutional neural network (CNN) approach. We conducted an experimental study on different attentional tasks for 46 children (ASD *n*=20, typically developing children *n*=26) and explored the limits of the face-based attention recognition model for participant and task differences. Our results show that the geometric feature transformation using an SVM classifier outperforms the CNN approach. Also, attention detection is more generalizable within typically developing children than within ASD groups and within low-attention tasks than within high-attention tasks. This paper highlights the basis for future face-based attentional recognition for real-time learning and clinical attention interventions.

**Supplementary Information:**

The online version contains supplementary material available at 10.1007/s41666-021-00101-y.

## Introduction

Researchers in autism spectrum disorders (ASD) fields have attempted several techniques to improve attention assessment for effective learning outcomes. Attention involves the cognitive and behavioral processing of discrete information while ignoring other information [1]. It is also described as the behavioral engagement [2] or cognitive engagement [3] of participants in a learning task. Children with ASD have challenges with attention as they are easily distracted away from learning tasks. ASD is a neurodevelopmental disorder with deficits in social communication and repetitive patterns of behavior [4]. The prevalence of ASD is on the increase, and it varies across countries. For example, the Centers for Disease Control and Prevention (CDC) of the USA reported 1 in 54 children in 2020 based on 2016 data as compared to 1 in 68 children in 2014 [5]. Attention deficit in children with ASD contributes to their low academic performance as compared to typically developing (TD) children [6]. Video data analysis is a common strategy for attention assessment. This analysis requires subjective annotation of observed attentional behaviors of participants at the end of a learning session. As a result, the method makes real-time attentional support impossible for children with ASD. Also, this assessment method is tedious and requires people with high expertise in ASD fields [7–9].

In recent studies, the dynamics of attention assessment has shifted from manual techniques to automated approach due to advances in sensor technologies and computer vision [10, 11]. One of the critical benefits of automated attention assessment is adaptive learning [12]. Some of the automated techniques used in previous studies include signal data from the brain [13], blood flow and heart rate [14], eye-tracking [15], galvanic skin conductance [16], and face-tracking [17]. Among these methods, face-tracking is the most promising approach because it is ubiquitous, cost-effective, and obtrusive [18–20]. This method is mostly implemented with a web camera or integrated camera in mobile devices. Due to these reasons, this paper focuses on the face-based attention recognition approach.

Automatic detection of facial landmarks has led to the development of applications that cut across different domains. Examples include where facial expression serves as intelligent systems for pain detection [21], syndrome diagnosis in newborns [22], driver’s drowsiness [23], emotion recognition [24], and engagement detection [2, 25]. The state-of-art of face-based attention or engagement recognition is usually centered on three stages: face detection (data collection and exploration), facial feature extraction, and classification [26]. The procedures of facial feature extraction can be divided into geometric and appearance-based approaches [27, 28]. In the geometric-based method, the facial features rely on the distances between specific facial landmarks. Yet, the appearance-based method is related to the pixel values obtained from facial texture, such as wrinkles, bulges, and furrows.

The appearance-based features provide crucial information for engagement detection. For instance, the work in [25] applied facial features to detect learners’ engagement through three different states; affective, cognitive, and behavioral. The authors gave graduate students an educational game about physics and coded the actions of the participants for on-task and off-task behavior. The findings from their study showed that the area under the receiver operating characteristic curve (AUC) for off-task detection was above chance (AUC = 0.816) for a generalized model. Additionally, the authors investigated the generalizability of the face-based model to temporal and demographic information of the participants. They found that the model could generalize across temporal and demographic information.

Another study used the appearance-based method to recognize student engagement from facial features [2]. The students played cognitive skills training software, and a Logitech web camera was used to record the entire session. The authors used a team of labelers consisting of undergraduate and graduate students from computer science, cognitive science, and psychology. These labelers viewed the recorded videos and labeled the learner’s facial appearance into four levels of engagement: not engaged, nominally engaged, engaged, and very engaged. The study findings revealed that the support vector machines (SVM) classifier had the highest performance score among the classifiers for the generalized model (AUC = 0.729). The authors evaluated how a face-based model generalizes across demographic factors (such as ethnicity). The model was trained with Caucasian and Asian-American populations and tested on an African American dataset. They found that the model generalized across ethnicity, and the prediction were above chance (AUC = 0.691).

In the geometric-based features technique, the key process relies on the Active Appearance Model (AAM) to track the facial landmarks. The localization of these landmarks is then used to track the distance variations in the face regions such as nose, eyes, and mouth to form feature vectors [29]. Geometric-based features are facial representations that aim to incorporate the knowledge from cognitive science to analyze temporal variation with respect to muscular activity [30]. The lengths of distances estimated from all pair distances are compared between posed emotions and neutral faces. The differences in the geometric distances obtained with respect to muscular activity are used to describe configural information of the face. Geometric-based features are commonly applied in two types of feature extraction: frame-based and sequence-based [31]. In the frame-based method, the geometric features use the distance between landmarks to represent the shape of facial components [32], while the geometric features in the sequence-based method capture the displacement information between the current frame and initial frame [29]. The sequence-based method applied in this study utilizes geometric features from 52 facial landmarks using SVM and multi-class AdaBoost for facial expression recognition. The geometric-based features technique can be applied to manual or automatic localized facial landmarks. Kotsia and Pitas [33] used only geometrical information on manually localized facial landmarks to detect facial expressions without taking into consideration any facial texture information. The distance variation between the first frame and the greatest facial expression intensity frame in a video was fed into a multi-class SVM classifier and achieved 99.7% accuracy. The method in [34] applies geometric-based features using AAM on a set of facial landmarks that are independent of head pose variations to recognize facial expression. The authors also applied a coupled scale Gaussian process regression model is used for normalizing head-pose. The application of geometric-based features in [35] took a step further by introducing stereo AAM to improve landmark-tracking by using a series of cameras to model 3D shapes. The authors of this study applied a generalized discriminant analysis classifier to merge 3-D shape and 2-D appearance for facial expression detection.

Unlike the appearance-based method, geometric features are computationally simple as it relies on basic operations [30, 36]. The work in [24] applies geometric-distance and angles between facial landmarks for engagement recognition using basic emotions and emotional transition in children with high functioning ASD. The model developed by the authors achieved 98.2% accuracy using the SVM learning algorithm with fewer features. Other studies on facial feature extraction have also adopted a geometric-based method for emotion detection. For example, the emotion recognition model in [37] uses geometric-based distances extracted from 11 facial landmarks around the eye, mouth, and jaw as facial features. The authors achieved an average recognition rate of 91.3%. Similarly, the method in [38] combines geometric features and angles between the facial landmarks. Their detection accuracy achieves 95.1%. The method developed in [39] combines the normalized distances around the eyes, nose, and mouth with the slope line segment from the facial landmarks as additional features and achieves an accuracy of 87.1%. The approach in [40] uses all the distances extracted from 83 facial landmarks as features with the probabilistic neural network (PNN) classifier algorithm, and the accuracy achieved was 90.2%. The studies highlighted in the previous paragraphs show the popularity and efficiency of SVM in recognizing facial expression using appearance-based and geometric-based features that were manually generated.

Aside from the manual feature extraction techniques for facial expression recognition, researchers also explore deep learning methods to extract facial features automatically [41, 42]. Convolutional neural network (CNN) is an example of deep learning commonly used for automatic facial feature extraction from images [43]. The CNN approach uses an end-to-end method where it selects the features to learn by itself during the training step. One of the main advantages of CNN is the capability of learning directly from input images and reducing the dependence on manual feature extraction techniques [44]. A study by Hua et al. [45] applies CNN for emotion recognition by extracting geometric facial features such as eyes and mouth from static input images, and they achieved 96.44% accuracy. Another study by Wu and Lin [46] uses CNN to extracted features from selected geometric areas of the face and achieved 96.27% accuracy. However, other studies reported lower recognition accuracy of 55.60% [47] and 54.56% [48] due to small data size. A recent review on facial recognition by Canedo and Neves [49] illustrated that traditional classifiers such as SVM could overcome the problems of using CNN classifiers on a small dataset to minimize overfitting [50]. The authors also discussed that a good understanding of a classification problem, proper pre-processing, feature selection, and hyperparameter-tuning could enhance traditional classifiers to achieve competitive results. In addition, feature transformation using the facial landmarks outputted by the face detector to calculate the distances between relevant facial landmarks can reduce noise on generated images during face detection.

The existing studies discussed in this section apply manual feature extraction (e.g., appearance-based and geometric-based features), as well as automatic feature extraction using the CNN approach. These studies show the potential of facial features for facial expression recognition in the TD population. Exploring the two methods of facial feature transformation in children with ASD can be an important research area as they express their emotions differently than TD children. Also, studies on appearance-based features explore the limitation of a generalized model with good detection accuracy for a typical population. Understanding how geometric-based feature extraction and transformation in children with ASD can reveal the limits of a generalized model. Additionally, these studies show the potential of manual labeling for observable affective expression and emotions. Nonetheless, manual labeling may lead to the loss of informative datasets due to inconsistency and ambiguity in labeling techniques [12]. However, combining observable attentional behavior with cognitive processing (i.e., performance test score) can reduce ambiguous labeling. This application of this manual labeling for attention in children with ASD might be ambiguous as well due to heterogeneity in the population.

In the current paper, we investigate two methods for attention recognition based on facial expression. The first is based on geometric feature transformation using an SVM classifier, and the second is based on the transformation of time-domain spatial features to 2D spatial images using a CNN approach for automated feature extraction and classification. We apply a distance threshold method for the geometric feature transformation to enhance the classification accuracy. The threshold method measures the differences between geometric distances of facial landmarks labeled as attention and inattention. In contrast to previous work, this paper explores the limits of model generalization across children with ASD. Furthermore, this paper considers observable attentional behaviors with performance test scores for labeling attention and inattention. The contributions of this work can be summarized as follows:

- Identification of geometric facial features that distinctively differentiate attention from inattention.
- Identification of the limitations of a generalized face-based attention recognition model.
- Identification of five prominent face regions for attention recognition.

The remainder of this article is organized as follows: Section 2 describes the methods used for data collection and model construction. Section 3 presents the results on model performance and how it generalizes across attention tasks and different demographic information such as the severity of ASD and TD. Section 4 presents the results of the study. Section 5 discusses the findings and identifies the challenges of face-based features for attention assessment. Finally, Section 6 concludes the research and highlights the direction for future work.

## Modeling Face-Based Attention Recognition

This section discusses the three stages of the proposed attention recognition methods: experimental setting and data collection, feature extraction stage, and classification stage.

### Experiment Setting and Data Collection

In the experimental stage, approval was obtained from the institutional review board committee of Qatar Biomedical Research Institute-Institutional Review Board approval was obtained before the commencement of the study. A total of forty-six children between the age of seven and eleven years participated in the study. Twenty children with ASD (ASD *n* = 20, *M* = 8.57, SD = 1.40) and Twenty-six TD children from the same age range (TD *n* = 26, *M* = 8.58, SD = 1.36) participated in the experiment. The ASD group had sixteen boys and four girls with mild to moderate ASD, while the TD group had eighteen boys and eight girls, as shown in Table 1. The ASD participants were recruited through an autism school in Doha and the Qatar Autism Society. All the ASD participants were clinically diagnosed by medical practitioners using the DSM-IV-TR criteria [51]. The TD participants were recruited from mainstream schools. Before conducting the study, the parent of the participants is asked to sign an informed consent form and fill the childhood autism spectrum test (CAST) questionnaire [52] to further identify the differences in the participants. According to the CAST questionnaire, participants who scored more than 15 out of the 32 questions among the TD participants may require further tests for ASD. Further experimental validation steps we took to achieve high data quality preventing the participant from eating or drinking during the experiment. The experiment was conducted in a quiet and dimly light room to avoid distraction and reduce illumination.

**Table 1:** Demographics of participants with ASD and TD group

Group	ASD (*n*=20)	TD (*n*=26)
Age	8.57 (1.40)	8.58 (1.36)
ASD moderate (mild)	11 (9)	-
CAST score	17.75 (2.04)	5.7 (3.2)
Gender: male (female)	16 (4)	18 (8)

**Fig. 1:**
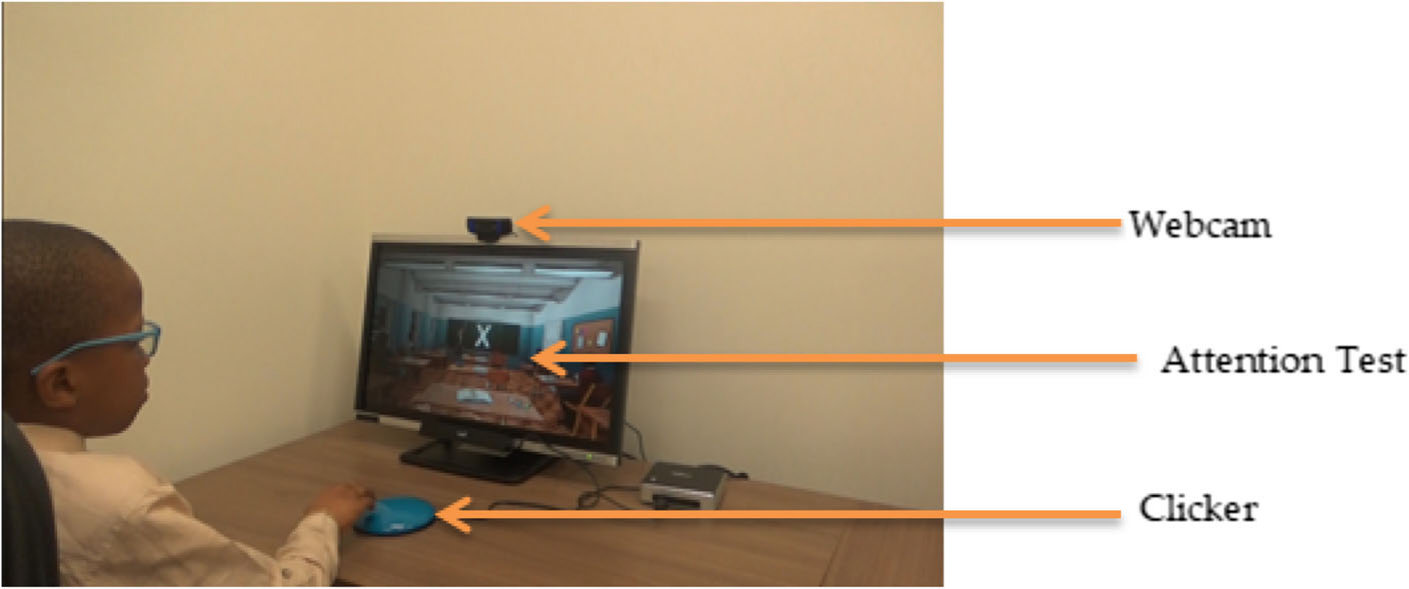
Real-time face-tracking during the attention task

All the participants took the attention tasks that simulate the continuous performance task (CPT) in a virtual classroom where the target stimuli were displayed as random alphabets on the board [25]. The random alphabets consist of target letters and other alphabets as well as classroom distractions. The test had four levels of distractions: baseline (no distractions), easy, medium, and hard. The participants were instructed to press the clicker when the target letter appears while we capture the participant with a webcam, as depicted in Fig. 1.

Attention behavioral rules from previous studies such as looking at the target stimuli and others [26] listed in Table 2 were used to generate class labels: attention and inattention. The video stream of the experiment was divided into separate time slots for each random letter displayed. Each letter appears for the duration of 1400ms. Then each slot was labeled as attention and inattention for correct (X-click) and incorrect click (Missed X), respectively. The total observation samples were extracted from 95 videos of all the participants. Each length of the video was 300 s long, and iMotions software [53] reads the video stream at 16 frame rate per second. The frames were annotated as attention and inattention using the software. The iMotions software generates 16 data samples in 1 second. During level 1(without distractions) of the attention test, 9607 and 9676 samples were obtained from ASD and TD groups respectively and models from each group were compared. Other observation samples generated from levels 2–4 (with distractions) in the ASD group were 25,495 samples.

**Table 2:** Observation checklist for attention and inattention annotation

Attention	Inattention
Participants looked at the screen and clicked the keyboard when letter X appears	Participants looked away and clicked the keyboard when letter X appears
Participants looked at the screen and called the letters on the screen.	Participants looked at the screen and did not click the keyboard when letter X appears.
	Participants looked at the screen and did not call the letters on the screen.
	Participants did not look at the screen and call the letters on the screen.

### Feature Extraction

**Fig. 2:**
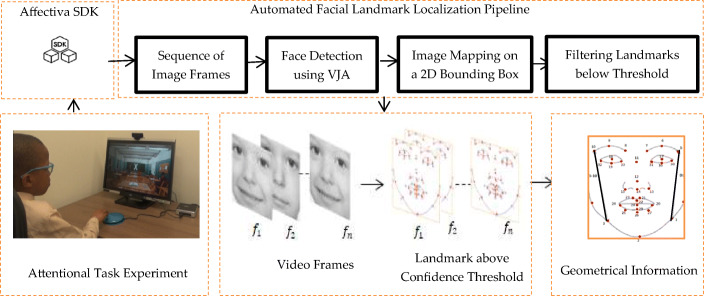
Block diagram of geometric-based feature extraction

The feature extraction stage describes the landmark localization and geometric-based feature extraction method used in deriving the geometrical information from the face (Fig. 4). The iMotions software embedded with Affectiva Software Development Kit (SDK) tracks and localizes facial landmarks (x and y coordinates), which are independent of head pose. Affectiva SDK automatically detects the face using the Viola-Jones face detection algorithm (VJA) [54] to detect 34 facial landmarks from a 2D video that are mapped onto a 2D image in a bounding box for each frame in the video (Fig. 2). Due to the drawback of 2D coordinates, head-pose and illumination variations, the SDK sets a threshold limit such that if the confidence of the landmark detection is below the threshold, then the bounding box and landmarks are ignored [55]. Thus, facial landmark coordinates will be missing at those frame instances with head pose invariant, and this preserves the quality of the geometric-base features. As a result, this software has shown a high percentage of accuracy when tested on over a set of 10,000 faces [56]. We used iMotions software to automatically extract 34 facial landmarks along with other features such as facial action units, emotions, and eye-tracking measures. Only the application of landmark features is reported in this paper. There are other types of open-access software that efficient for the automatic detection of facial landmarks, such as OpenCV, Dlib, Open Face, and others. We chose iMotions as it provides us with a robust approach of obtaining different features simultaneously and manually annotating video frames with desired features or anomalies. Previous studies in the facial detection domain also use similar software to extract 2D video for emotion recognition [57–60].

**Fig. 3:**
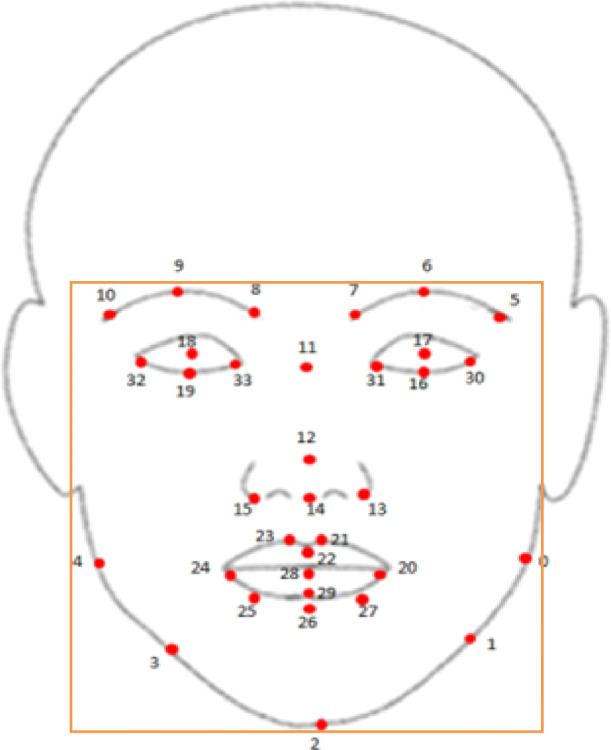
Thirty-four facial landmarks with labels. Where 0, right top jaw; 1, right jaw angle; 2, gnathion; 3, left jaw angle; 4, left top jaw; 5, outer right brow; 6, right brow corner; 7, inner right brow corner; 8, inner left brow corner; 9, left brow center; 10, outer left brow corner; 11, nose root; 13, nose lower right boundary; 14, nose bottom boundary; 15, nose lower left boundary; 16, outer right eye; 17, inner right eye; 18, inner left eye; 19, outer left eye; 20, right lip corner; 21, right apex upper lip; 22, upper lip center; 23, left apex upper lip; 24, left lip corner; 25, left edge lower lip; 26, lower lip center; 27, right edge lower lip; 28, bottom lower lip; 12, nose tip; 29, top lower lip; 30, upper corner right eye; 31, lower corner right eye; 32, upper corner left eye; 33, lower corner left eye

Given a face with 34 landmarks that cover eyes, eyebrows, nose, lips, and jaw described in Fig. 3, we extracted a pool of feature vectors represented in Eq. 1. Suppose ${\mathrm{f}}_{\mathrm{n}}^{\mathrm{i}}$ denotes each landmark in the *n*th video frame, starting with the *i*th frame.

1 $$ {f}_n^i=\left[\begin{array}{ccc}{x}_{0,}^i{y}_0^i\kern0.75em {x}_{1,}^i{y}_1^i& \cdots & {x}_{33}^i{y}_{33}^i\\ {}\vdots \kern3.25em \vdots & \cdots & \vdots \\ {}{x}_{0,}^n{y}_0^n\kern0.75em {x}_{1,}^n{y}_1^n& \cdots & {x}_{33}^n{y}_{33}^n\end{array}\right] $$

Aside from the facial representation using raw coordinates of the landmarks, the distances between facial landmarks can give further description about the temporal variation of muscular activity. The lengths of distances estimated from all pair distances are compared between posed and neutral faces are used for emotion recognition [24, 29, 30, 61]. We transformed the facial landmark coordinates into geometric-based features measured in millimeters (mm). The geometrical information is estimated for each frame in the video using the Euclidian distance formula in Eq. 2.

2 $$ \left[\left({x}_1,\kern0.5em {y}_1\right),\left(\ {x}_2,\kern0.5em {y}_2\right)\right]=\sqrt{{\left(\ {x}_2\hbox{--} {x}_1\right)}^2+{\left(\ {y}_2\hbox{--} {y}_1\right)}^2\ } $$

where x_1_, y_1_ and x_2_, y_2_ are representing two different facial landmarks. The estimated geometrical information generated was between one landmark to other landmarks, and these sums up 560 geometric-based feature distances. One way to further ascertain minimal error of landmark distortion due to head pose and illumination is to estimate Mean Absolute Error (MAE) between predictions and ground truth [62–64]. We estimated the MAE in Eq. 3 between two consecutive frames in the frame-based geometric features for each participant. We examined the distance between two landmarks that are sensitive to distortion: right top jaw and left top jaw for the two-class label.

3 $$ \mathrm{MAE}=\frac{1}{n}{\sum}_{i=1}^n\mid {y}_i-{x}_{i\kern0.5em }\mid $$

where *n*=total number of frames, *y*_*i*_ denotes initial frame, and *x*_*i*_ is the subsequent frame.

### SVM Classification Algorithm

In this section, the feature selection process and the SVM classification algorithm are discussed. The SVM algorithm maximizes the margin between training data of two different classes separated by a hyperplane (decision boundary) [65]. The hyperplane can either be linear or complex, depending on the distinctiveness of the classes. A linear hyperplane is used when the classes are linearly separable; a linear kernel decides the boundary between the two class labels. In the case of attention recognition, a linear hyperplane cannot accurately separate attention and inattention as the facial features of these classes are closely related, and difficult to decide a linear decision boundary. A complex separating hyperplane efficiently separates two classes that are not linearly separable using a Gaussian radial basis function (RBF). RBF implements a non-linear kernel to make the classes more separable. A kernel is a function that maps a non-linear hyperplane into a higher-dimensional space in which makes classes linearly separable. Studies on face-based classifier algorithms reported that SVM with a non-linear kernel was the most accurate among other classifier algorithms [2, 24, 66]. There are two main parameters of the kernel that mainly influence the ability of an SVM to discriminate between two classes. One of the parameters is the class-specific penalty, *C*, which determines a decision boundary that misclassifies a percentage of training samples. A large value for *C* indicates the model will be stricter on misclassification errors. The other parameter, gamma (*γ*), influences the sophistication of the decision boundary. Small values of *γ* will lead to an increasingly sophisticated boundary that correctly classifies a higher percentage of training data. Thus, inappropriate value selection for these parameters, also known as hyper-parameter tuning, may lead to the poor performance of a model on a new dataset (i.e., overfitting). The parameter values selected for *C* and *γ* are from the following sets of values *C* = [1–26] and *γ* = [0.001, 0.01, 0.1, 1, 10]. Based on the best cross-validation results, the final parameter values chosen were (*C* = 11 and *γ* = 0.1).

Selected features require standardization for most machine learning algorithms to prevent biased predictions. Samples with more significant variance usually dominate other samples with lower variance, and this prevents the algorithm from correctly learning all the features. Some of the algorithms (e.g., Gaussian SVM) assume samples have a similar variance to secure unbiased learning. The transformed features were standardized to ensure data sample range restriction and close to normal distribution. This technique subtracts the mean value of the samples and divides their value by the standard deviation, as shown in Eq. 4. Standardizing features results provide the mean of the distribution as 0, and the values are mostly between −1 and 1. This approach ensures that each feature contributes to a consistent ratio in the model prediction. Each sample of standardized feature vectors is labeled as attention or inattention for developing the model.

4 $$ Z=\frac{x-m}{s}, $$

where *Z* is the standardized score, *m* is the mean of the training samples, and *s* is the standard deviation of the training samples.

**Fig. 4:**
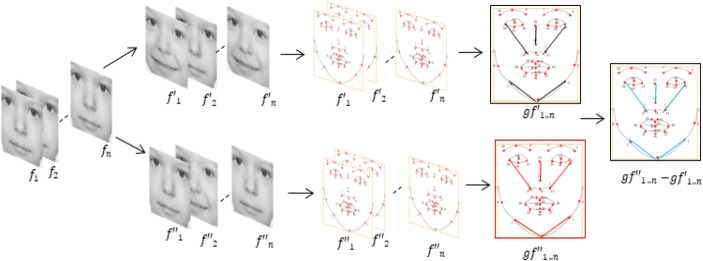
Feature selection process

The geometrical information distinguishing attention and inattention frames based on distance threshold value is used to boost the classification performance of the non-linear SVM algorithm. The distance threshold value is an established method for revealing the information embedded in a dataset [67]. This approach has been successfully applied in differentiating posed emotions from neutral faces [24, 68, 69]. In this paper, we used the following four steps to obtain the best geometrical information that distinctively separates attention and inattention class (Fig. 4).

**Fig. 5:**
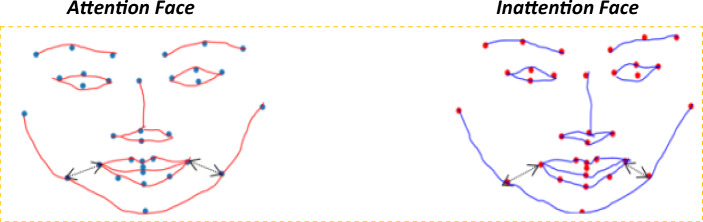
Mean intensity frame for attention and inattention

In the first step, we identified the frames that represent attention and inattention class by manually annotating video frames using the attentional behavior rules described in Table 4. Then, we use iMotions software to manually annotate the video frames into attention, inattention, and invalid frames where landmarks are aligned outside the participants’ face.where f_1. . n_ = frame by frame detection is, f′_1. . n_ = frames annotated as attention, f′ ′_1. . n_ = frames annotated as inattention, gf′_1. . n_ = geometrical information of attention, gf = geometrical information represented by mean value of landmark coordinates and gf ′ ′_1. . n_ − gf′_1. . n_ = the difference between the mean value of attention and inattention frames. In the second step, we estimated the geometrical information by connecting all points pairwise in space from the annotated frames using the Euclidean distance formula denoted by *d*(*ab*) (Eq. 5).

5 $$ d\left(\mathrm{a}b\right)=\sqrt{\sum_{i=1}^n{\left(\ {a}_i\hbox{--} {b}_i\right)}^2} $$

where *a* and *b* are representing two different facial landmarks. *d*(*ab*) represents the Euclidean distance between the landmarks. In the third step, we calculated the mean value for all the landmark coordinates in each class denoted by *f*_*g*_(mean) in Eq. 6. These features describe the differences in the componential information for attention and inattention as mapped from the raw data where the mean intensity of the attention face seems to be looking at the center of the screen and inattention to be looking sideways to the corner of the screen (Fig. 5)

6 $$ {f}_g\left(\mathrm{mean}\right)=\frac{1}{n}{\sum}_{i=1}^n{f}_i\left(x^{\prime }y^{\prime}\right) $$

In the last step, we estimated the difference between the mean value of attention class and in attention class denoted by gf in Eq. 7. Then, the threshold values that differentiate attention from inattention frames were sorted in descending order. The geometrical information with best highest threshold values was selected in the multiples of 10s to train the SVM classifier algorithm.

7 $$ gf= gf^{\prime }{\prime}_{1..n}- gf{\prime}_{1..n} $$

### CNN Network Architecture

CNN is a deep learning neural network method that is commonly used for image classification. CNN has an architectural structure that consists of two main parts: feature extraction and classification. The feature extraction part takes an image as an input, and it applies convolution layers with several kernel filters to extract features that are passed to an activation function, such as the rectified linear unit (ReLU) to increase nonlinearity in the network. The pooling layer then distills the output of the convolutional layer to simple and salient elements. The convolutional layers and max-pooling can be repeated as necessary. The extracted features are passed to fully connected layers, which compiles the extracted features from the previous layers to form the final output. The CNN model trains these features through forwarding- and back-propagation at different epochs until it achieves a distinct network with trained weights and features. Recent studies in different domains use CNN to solve complex problems such as image classification and object detection due to their enhanced performance compared to typical machine learning models [70, 71].

**Fig. 6:**
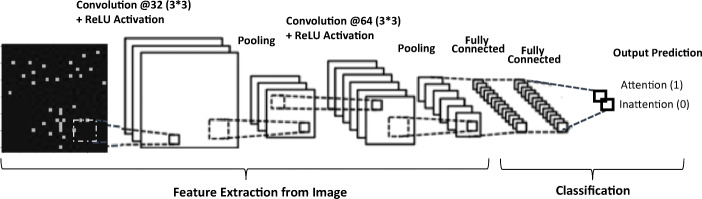
Attention classification using a CNN model structure

In this model, we applied a multi-channel and multi-layer CNN for our binary classification problem. The structure of the CNN architecture has an input image generated from time-domain spatial features (i.e., the frame by frame facial landmarks coordinates) to 2D spatial images with the size of 32 by 32 pixels. The facial landmarks are represented as white dots on a black background to reduce the noise of the image. These images went through two convolution layers of 32 and 64 feature maps using filters with a convolution kernel of a 3 by 3 receptive panel each. The model has two max-pooling layers with sizes 2 by 2 after every convolution layer. The fully connected layers have depths of 62 and 1. We added a 20% dropout to reduce the possibility of overfitting. The outputs of these networks were attention and inattention. This is illustrated in more detail in Fig. 6.

## Results

This section presents the findings on best facial features and face regions with the best distance threshold for attention recognition and model performance using used AUC and F1-score as the classification evaluation metric due to class imbalance. Next, the section compares the performance of the proposed face-based attention recognition model of participant-specific and participant-independent. Lastly, the limits of the generalized model are further investigated.

### Best Geometrical Information for Detecting Attention

The evaluation metrics used to evaluate the performance of the proposed models are accuracy (ACC) and AUC. These metrics were used for three different best-selected geometric-based features (i.e., 10, 20, and 30 features). The ACC of the models uses random data splits of the training and testing data (80% and 20 %) with 10 cross-validations without reference to participants. The AUC was evaluated using (n-1) participants for training and testing the last participant. The model with the best 20 features had highest performance score (ACC=0.889, AUC= 0.531) than best 10 (ACC=0. 873, AUC= 0.524) and 30 features (ACC=0. 873, AUC= 0.521). Thus, this study used a model with the best 20 distance-based features due to their higher performance and fewer features. Table 3 describes these 20 distance-based features. The distance threshold between all the feature vectors of attention and inattention identified the best distinctive features. The best features emerged by sorting the distance threshold values in descending order. They were selected to train the recognition model. Five face regions emerged as prominent features in recognizing attention—jaw, eyes, eyebrows, nose, and gnathion. A study by [24] highlighted similar findings with high-functioning ASD. The authors uncovered brow, nose, eyes, and lips as the best face regions for the transition of emotions among children with high functioning ASD during task engagement.

**Table 3:** Best 20 distance-based features used for the SVM algorithm

Features	Feature description	Inattention (mean values)	Attention (mean values)	Distance threshold values (mm)
D: 3–15	Left jaw angle-outer right brow corner	171.45	146.9	24.55
D: 4–5	Left top jaw-outer right brow corner	168.45	144.06	24.38
D: 4–6	Left top jaw-right brow center	149.17	125.72	23.45
D: 3–6	Left jaw angle-right brow center	158.32	135.01	23.30
D: 4–16	Left top jaw-outer right eye	148.79	126.22	22.56
D: 4–7	Left top jaw-inner right brow corner	122.89	100.99	21.89
D: 4–31	Left top jaw-lower corner right eye	132.7	110.85	21.85
D: 4–30	Left top jaw-upper corner right eye	134.16	112.37	21.79
D: 0–4	Gnathion-outer right brow corner	167.05	145.36	21.69
D: 3–16	Left jaw angle-outer right eye	147.11	125.43	21.67
D: 2_5	Gnathion-outer right brow corner	159.94	138.36	21.58
D: 3_7	Left jaw angle-inner right brow corner	136.43	114.93	21.49
D: 3_31	Left jaw angle-lower corner right eye	131.48	110.45	21.02
D: 3_30	Left jaw angle-upper corner right eye	136.74	115.77	20.96
D: 4_13	Left top jaw-nose lower right boundary	111.63	90.99	20.63
D: 4_12	Left top jaw-nose tip	95.09	74.58	20.51
D: 4_17	Left top jaw-inner right eye	117.02	96.6	20.42
D: 4_11	Left top jaw-nose root	98.58	78.51	20.06
D: 2_6	Gnathion-right brow center	154.53	134.66	19.87
D: 3_17	Left jaw angle-inner right eye	121.38	101.61	19.77

### Model Performance

**Fig. 7:**
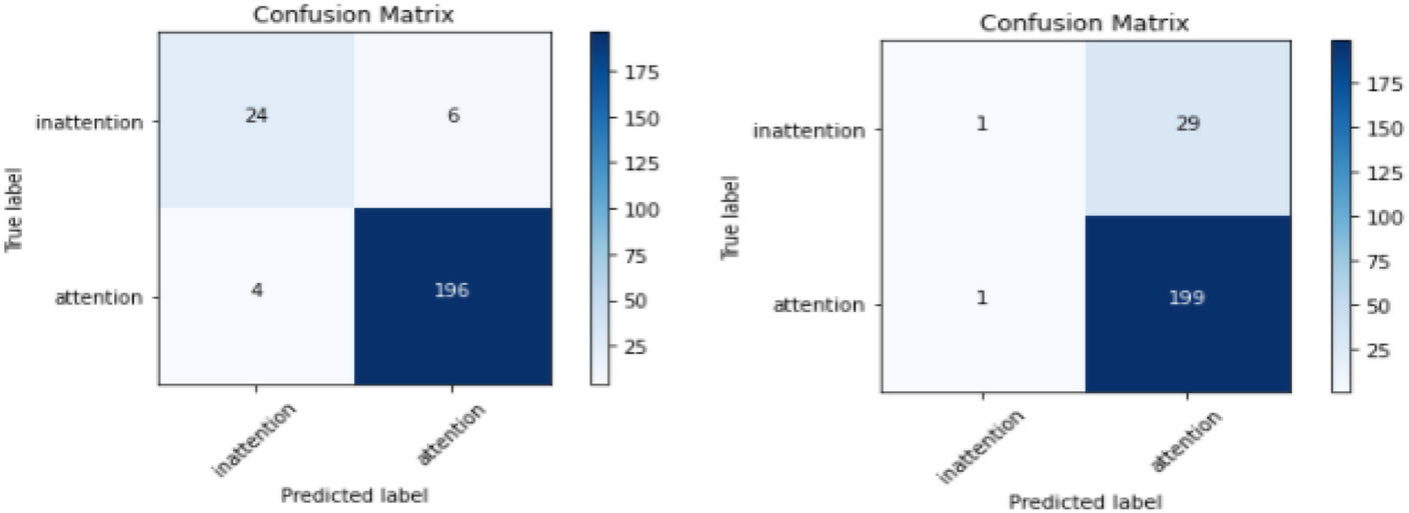
Confusion matrix for participant-specific model for P1 (left: SVM; right: CNN)

The participant-specific model was trained and tested only on the data sample from each participant. This model type detects attentional behavior specific to each participant. Building a participant-specific model, especially for children with ASD, is imperative due to heterogeneity in the spectrum [72]. The attention recognition model was trained and tested only on the data sample from each participant in the two proposed models: SVM and CNN. The ratio of training and testing data was 80% to 20% for each participant, and model performance was averaged across all participants. The averaged training and testing for the SVM model achieved a higher performance score for training (ACC=0.995) and testing (ACC=0.959, AUC=0.965) than the CNN model for both training (ACC=0.944) and testing (ACC=0.894, AUC=0.856) as shown in Table 4. The confusion matrix evaluation of the two models illustrated for a participant (P1) shows that SVM only misclassified 6 out of 30 inattentional samples as attention while CNN misclassified 29 out of 30 inattentional samples (Fig. 7). Conversely, CNN misclassified 1 out of 200 attentional samples as inattention while SVM misclassified 4 out of 200 attentional samples. This shows that the CNN approach can recognize attentional behavior more than the SVM approach and vice versa for inattentional behavior. Overall, both methods imply that attention recognition among children with ASD can be achieved with facial features. Nonetheless, the SVM approach shows better performance than the CNN approach.

**Table 4:** Evaluation of participant-specific model using SVM and CNN

	SVM			CNN		
	Training	Test		Training	Test	
	ACC.	ACC	AUC	ACC	ACC	AUC
P1	1.000	0.957	0.941	0.965	0.900	0.767
P2	0.998	0.997	1.000	0.987	0.972	0.850
P3	0.998	0.952	0.992	0.970	0.800	0.822
P5	0.999	0.984	0.904	0.998	0.974	0.915
P6	0.996	0.989	0.996	0.978	0.951	0.886
P7	0.997	0.920	0.970	0.829	0.720	0.826
P8	0.993	0.945	0.983	0.944	0.895	0.835
P10	0.994	0.984	0.878	0.990	0.982	0.856
P12	0.950	0.920	0.941	0.971	0.842	0.828
P13	1.000	0.996	0.964	0.994	0.956	0.888
P14	0.997	0.839	0.929	0.981	0.718	0.760
P15	0.998	0.972	0.978	0.948	0.889	0.918
P16	1.000	0.954	0.992	0.767	0.903	0.966
P18	0.992	0.939	0.981	0.958	0.935	0.941
P19	1.000	1.000	1.000	0.831	0.885	0.789
P20	1.000	0.995	0.995	0.990	0.982	0.856
Avg.	0.995	0.959	0.965	0.944	0.894	0.856

**Fig. 8:**
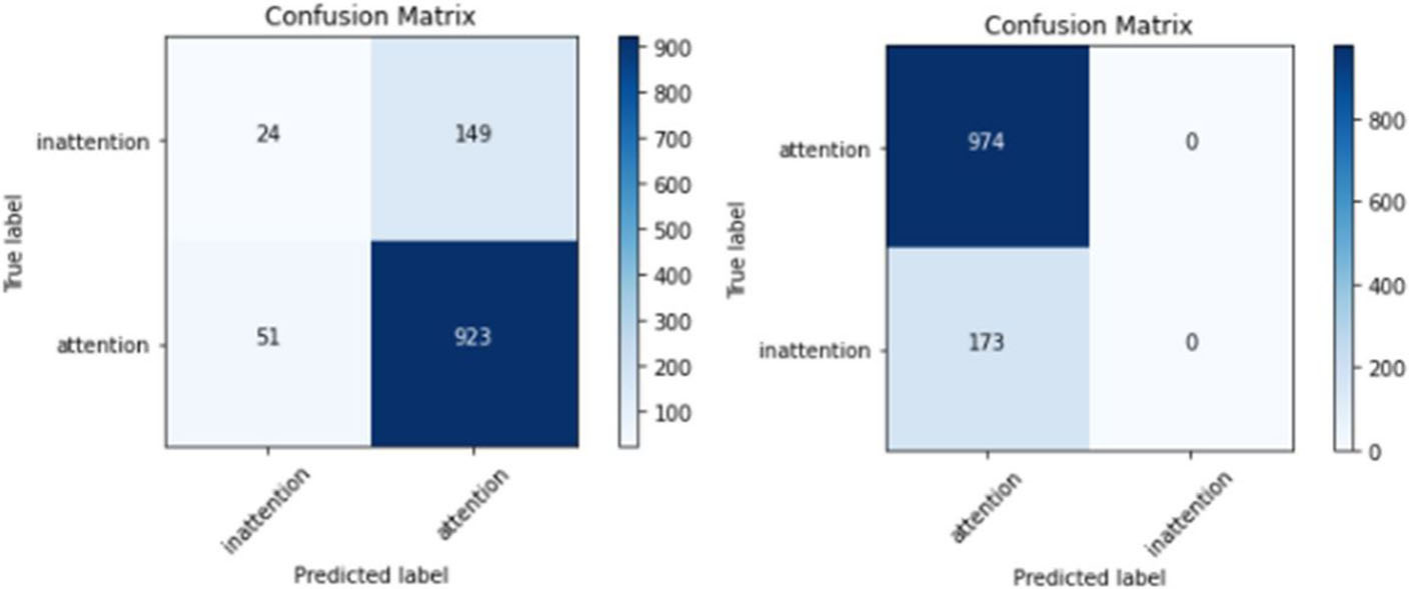
Confusion matrix for generalized model for P1 (left: SVM; right: CNN)

In the generalized model evaluation, we used the leave-one-out model evaluation. The model was trained on all the participants and tested on a new participant who was not part of the training data. The model performance average for all participants using the SVM and CNN models were compared to identify the model that suits our objective. The SVM model achieved average training higher performance score (ACC= 0.956) but a lower testing performance score (ACC=0.7 15, AUC=0.536) than the CNN model training performance score (ACC=0.884) and testing performance score (ACC=0.808, AUC=0.591) as shown in Table 5. The confusion matrix evaluation of the two models illustrated for a participant (P1) in Fig. 8 shows that SVM misclassified 146 out of 173 inattentional samples as attention while CNN misclassified all the 173 inattentional samples as attention. In contrary, CNN did not misclassify any attentional samples, but SVM misclassified 51 out of 974 attentional samples as inattention. This shows that the SVM approach still performed better in recognizing inattentional behavior more than the CNN approach and vice versa for attentional behavior. Overall, both methods could recognize attentional behavior more than inattentional behavior. Nonetheless, the SVM approach could relatively recognize in attentional behavior than the CNN approach.

**Table 5:** Evaluation of participant-specific model using SVM and CNN

	SVM			CNN		
	Training	Test		Training	Test	
	ACC.	ACC	AUC	AUC	ACC	AUC
P1	0.958	0.825	0.532	0.894	0.849	0.551
P2	0.954	0.936	0.288	0.878	0.968	0.678
P3	0.951	0.905	0.315	0.875	0.766	0.581
P5	0.957	0.683	0.654	0.873	0.936	0.597
P6	0.954	0.874	0.271	0.886	0.936	0.519
P7	0.959	0.595	0.584	0.893	0.609	0.469
P8	0.956	0.724	0.593	0.878	0.884	0.568
P10	0.954	0.668	0.594	0.872	0.976	0.334
P12	0.956	0.302	0.688	0.891	0.824	0.535
P13	0.956	0.833	0.578	0.884	0.93	0.597
P14	0.954	0.877	0.586	0.913	0.546	0.425
P15	0.958	0.390	0.577	0.885	0.775	0.597
P16	0.955	0.839	0.676	0.886	0.906	0.768
P18	0.956	0.765	0.388	0.873	0.928	0.735
P19	0.955	0.577	0.665	0.894	0.106	0.667
P20	0.957	0.645	0.590	0.866	0.984	0.837
Avg.	0.956	0.715	0.536	0.884	0.808	0.591

### Limits of Model Generalization for Children with ASD

This section discusses the limits of model generalization across children with ASD. It explores how a model trained on ASD group generalizes to children within and outside the spectrum and how it generalizes across attention tasks with distractions. The number of participants from the ASD group who completed the experiment was lower than the TD group (ASD = 18, TD = 25). Hence, the number of participants from the TD group was reduced to 18 to match the ASD group. The total number of participants considered for the model generalization was 36 from both groups.

**Fig. 9:**
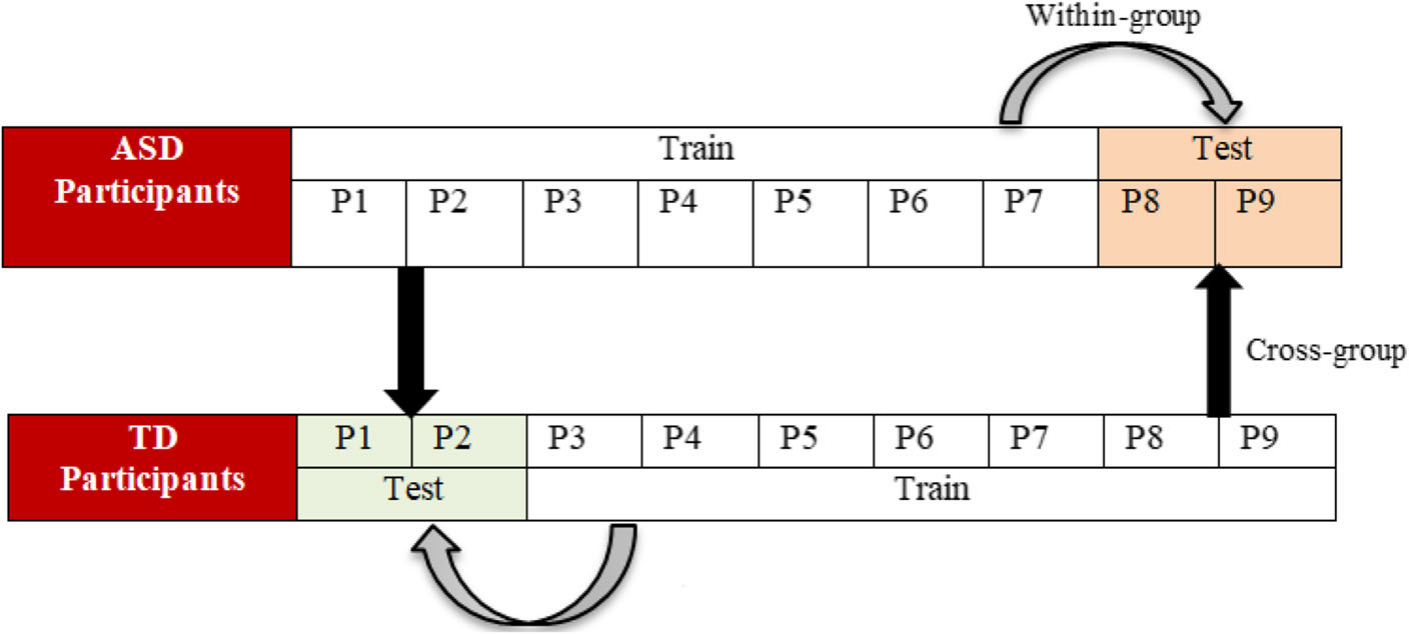
Example of cross-groups and within-group with the participant–independence level

This study used bi-directional cross-group model evaluation as well as within-group evaluation to gain insight into variations in attention behavior across groups of participants. The analysis of the model performance is discussed using AUC and F1-score for a bi-directional cross-group and within-group model evaluation due to imbalanced data [73]. While AUC handles the imbalance data from two directions in a binary class classification, F1-score works well only in one direction [19]. We applied the SVM approach for this evaluation due to its relatively higher recognition for both attention and inattention than the CNN approach for both participant-specific and generalized models. The data were partitioned into training and testing sets at a ratio of 80% to 20%. For example, Fig. 9 illustrates the two-way splitting pattern between ASD and TD participants using several iterations as recommended in previous studies [18, 20]. This paper considered 50 iterations for all the cross-model evaluations.

**Fig. 10:**
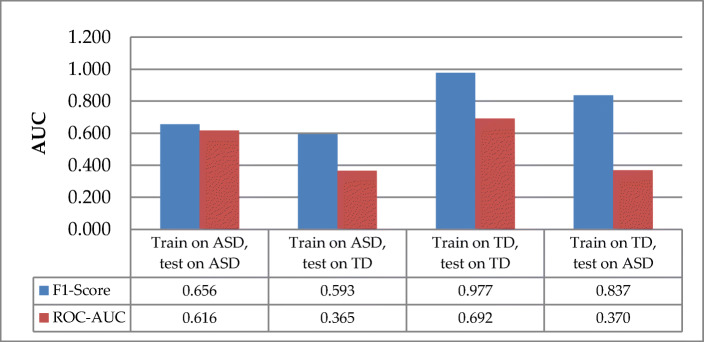
Model generalizations between children with ASD and TD

Figure 10 illustrates the evaluation of model generalization between ASD and TD groups using two metrics: F1-score and AUC. The F1-score shows that the model generalizes more in TD (F1-score = 0.977) than in the ASD group (F1-score of 0.656). The F1-score dropped from within-groups to cross-group models for ASD and TD with a percentage value of 14% and 6%, respectively. This percentage difference indicates that generalizing the ASD model to TD participants is less efficient than the other way around. Similarly, the AUC metrics show that the within-group model evaluation shows that the TD group (AUC = 0.692) performed better than the ASD group (AUC = 0.616). In the cross-group model, testing the ASD model with TD data (AUC = 0.365) gave less performance than testing the TD model with ASD data (AUC = 0.370). Additionally, the performance of the cross-group model dropped was lower as compared to the within-group model. The decrease in model performance from within-group to cross-group showed that each group exhibits different attentional behaviors. Also, the model performance was above chance only for within-group evaluation and not for cross-group assessments. The performance of the within-group model indicates that the model only generalizes for within-group, not cross-groups.

**Fig. 11:**
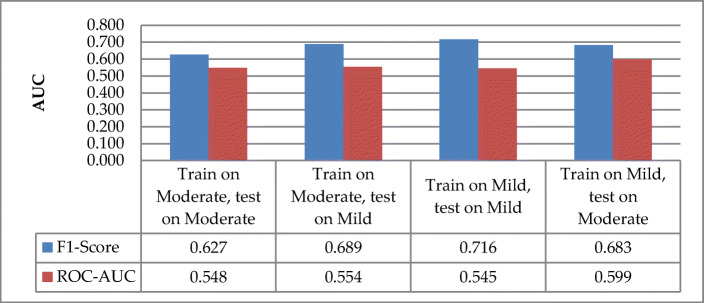
Model generalization between children with mild ASD and moderate ASD

According to the F1-score and AUC metrics presented in Fig. 11, the F1-score of model generalization between moderate ASD and mild ASD shows that the model generalizes within the mild ASD group (F1-score = 0.716) than within the moderate group (F1-score = 0.627). The cross-groups model shows that training moderate ASD and testing with mild ASD has better performance (F1-score = 0.689) than the model in the opposite direction (F1-score = 0.627). The model performance increases from within-group to cross-group only for the moderate ASD group with 6.2% and decreases for the mild group with 3.3%. In contrast, the AUC evaluation shows that the attentional recognition model for within-groups mild ASD group (mild: AUC = 0.545, moderate: AUC = 0.548) and cross-groups (mild-moderate: AUC = 0. 599, moderate-mild: AUC = 0. 554) are slightly above chance level. The cross-group model’s performance was better than that of the within-group by 0.6% (moderate-mild) and 5.4% (mild-moderate). The slight increase in performance shows that attentional behaviors in mild ASD generalize more to moderate ASD than in the opposite direction.

**Fig. 12:**
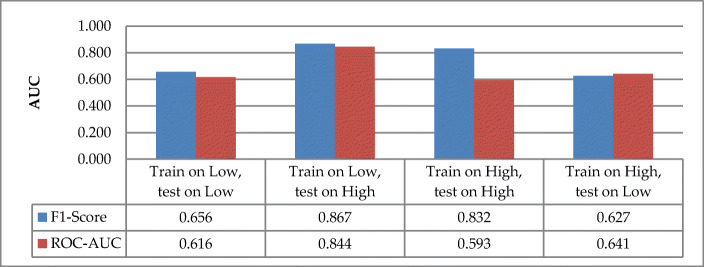
Model generalizations between children with ASD and TD

The evaluation of the F1-score metric for model generalization between tasks for children with ASD shows that the model for high distraction tasks (F1-score = 0.832) generalizes more than low distraction tasks (F1-score = 0.656) as shown in Fig. 12. The cross-task model shows that training low distraction tasks and testing for high distraction tasks led to better performance (F1-score = 0.867) than the other way around (F1-score = 0.627). The model performance increases from within-task to cross-task only for low distraction tasks with 21.1% and decreases for high distraction type with 20.5%. The percentage difference shows that attentional behaviors are more generalized for tasks with low distractions than tasks with high distractions. The second performance metric (AUC) shows that the within-task model for low distraction (AUC = 0.616) performed better than that of the task with high distraction (AUC = 0.593). The performance of the cross-task model illustrates that the model of a task with low distractions was better (AUC = 0.844) than that of a task with high distractions (AUC = 0.641). The model performance increases from within-task to cross-task in both attention-task types, with 12.8% in low distractions and 4.8% in high distractions. This percentage increase shows that attentional behaviors are better defined in tasks with low distraction than in tasks with high distraction.

### Evaluation of Landmark Distortion

The evaluation of landmark distortion due to head poses and lighting using MAE estimation between geometric-based features between right and left top jaw (D: 0–4). These features are prone to distortion due to pose variations. The estimated errors in Table 6 show that at all errors, values between these landmarks lie in the range between 0 and 1.06 mm. These error values across the participants indicate that there is minimal landmark distortion in the dataset.

**Table 6:** Evaluation of landmark distortion across participants (mm)

Name (ASD)	Error (Attn.)	Name (TD)	Error (Attn.)
P1	0.102	T10	0.035
P10	0.084	T11	0.085
P11	0.015	T12	0.087
P12	0.059	T13	0.136
P13	0.101	T14	0.041
P14	0.128	T15	1.010
P15	0.138	T16	0.531
P16	0.276	T17	0.156
P18	0.096	T18	0.165
P19	1.065	T19	0.183
P20	0.088	T2	0.059
P2	0.094	T3	0.199
P3	0.088	T4	0.033
P3	0.251	T5	0.101
P4	0.026	T6	0.165
P5	0.096	T7	0.031
P6	0.094	T8	0.129
P7	0.508	T9	0.089

*ASD* autism spectrum disorder, *TD* typically developing, *Attn.* attention

## Discussion

This paper proposed two methods for attention recognition using facial expression. The first is based on geometric-based feature transformation using an SVM classifier, and the second is based on the transformation of time-domain spatial features to 2D spatial images using a CNN approach for automated feature extraction and classification. We developed a virtual classroom to elicit the attentional behaviors of the children with ASD and TD children in an ecologically valid classroom. iMotions software embedded with Affectiva SDK was used to automatically generate 34 facial landmarks with x and y coordinates in real time during the attention task. The recorded attention task session was manually annotated as attention and inattention based on the response of the participant to the target stimuli in the attention task. The facial landmarks data in the annotated video frames were used for the proposed methods.

In the first approach, the annotated landmarks generated were transformed into geometric-distance features, and a distance threshold method was applied to the geometric-distance features to gain further insight into attention recognition. The features with higher threshold values were used to train a non-linear SVM classifier to detect attention and inattention using the participant-specific and generalized models. The comparison of a participant-specific and generalized model using the SVM approach shows that the averaged training and testing for the participant-specific model achieved a higher performance score for both training and testing than the generalized model. The confusion matrix evaluation of the two models for a participant (P1) shows that the participant-specific model correctly classified 98% of attentional behavior and 80% of inattentional behavior. In contrast, the generalized model correctly classified 94.8 % of attentional behavior and 13.9% of inattentional behavior. These results illustrate that the SVM approach can recognize more attentional behavior than inattention. Similarly, attentional behavior can be more generalized than inattentional behavior, which indicates that inattentional behavior varies across participants.

The second approach used 2D spatial images generated from the annotated landmarks, which were fed into a multi-channel and multi-layer CNN for binary classification. The images went through two convolution layers and two max-pooling layers after every convolution layer and lastly through fully connected layers to output attention or inattention. Comparing the performance of the participant-specific and generalized model using the CNN approach, our results show that the averaged training and testing for the participant-specific model achieved a higher performance score than that of the generalized model. The confusion matrix evaluation of the two models for a participant (P1) shows that the participant-specific model correctly classified 99.5% of attentional behavior and 3.3% of inattentional behavior. In contrast, the generalized model correctly classified 100% of attentional behavior and 0% of inattentional behavior. These results illustrate that the CNN approach recognizes more of attentional behavior than inattention. Similarly, the attentional behavior can be more generalized than inattentional behavior, which shows the differences in how participants exhibit more different inattentional behavior than attentional behavior.

The analysis of the two proposed model design approaches shows that they can efficiently recognize attentional more than the inattentional behavior. The CNN approach shows higher recognition power for attentional behavior than the SVM approach in attentional behavior and vice versa for inattentional behavior. Overall, the comparison of the two approaches for recognizing attention and inattention. SVM approach shows relatively better performance than the CNN approach and in participant-specific than the generalized model. These findings reveal that the SVM approach using transformed geometric-based features with Euclidean distance can distinctively differentiate attention from inattention. This outcome is similar to the findings in [74], where the authors compare SVM and CNN in facial spoof detection. The SVM achieved an accuracy of 91%, outperforming the CNN approach, which only achieved 76.31%.

Lastly, we applied the SVM approach to explore how the model can be generalized across ASD and TD groups and the different attention tasks due to its higher recognition power in this study. The results from this approach show that the generalized model for attention recognition in children with ASD cannot be generalized for different participants and attention tasks. The model performance for ASD and TD groups shows the disparity of attentional behavior among children with ASD than in the TD group. This disparity may be associated with the heterogeneity in children with ASD as each child in the spectrum behaves differently than another [75, 76]. The two metrics: F1 score and AUC, show consistent results on model generalization for the within-group model for ASD and TD on attention tasks without distractions. Nonetheless, the model did not generalize across ASD groups for attention tasks with different degrees of distractions. This finding shows the limitation of model generalization across participants with ASD and tasks with different distraction levels. Thus, different attention tasks will also affect attention recognition model generalization in children with ASD. This result is attributed to the evidence-based study by Smith T and Iadarola [77] where the authors found that children with ASD show more attentional behaviors in attention tasks with high distractions than those with low distractions.

The three novel contributions in this paper include the identification of geometric facial features that distinctively differentiate attention from inattention, the limitations of a generalized face-based attention recognition model for children with ASD, and five prominent face regions in children with ASD for attention recognition. Despite these contributions, here are the limitations. (1) This study focused on the presence of ASD, its severity, and attention tasks. Atypical attention among children with ASD is not only determined by the effect of the severity of ASD on attentional support but also by age and gender. Studies have shown that females with ASD show less repetitive behaviors as compared to males [78, 79]. (2) The attention tasks used for data collection simulate sustained and selective attention, which may not apply to other forms of attention types such as joint attention and divided attention. 3) Although the attention tasks simulated the ecological validity of potential classrooms, findings in this study may be different from attentional behavior in real classrooms.

## Conclusion and Future Work

This paper proposed a face-based attention recognition model using two methods. The first is based on geometric feature transformation using an SVM classifier, and the second is based on the transformation of time-domain spatial features to 2D spatial images using the CNN approach for automated feature extraction and classification. A comparison of the two methods on model performance for participant-specific and generalized models shows that the former model has better performance. The evaluation of geometric feature transformation using an SVM classifier outperformed CNN in the participant-specific model, and the two algorithms show similar performance for the generalized model. The proposed method extends existing research on facial feature extraction and transformation for attention recognition as well as objective attention annotation of facial features during learning. This study also investigated how the geometric feature transformation-based model generalized to attention tasks, presence, and severity of ASD. In general, this paper shows that the proposed method was effective for recognizing attentional behavior that is unique to each child with ASD than across the spectrum. The recommendations for future work are further analyzing how the face-based attention recognition model generalizes to other demographic information such as age and gender. Additionally, a similar study should be conducted in a real classroom to compare the findings.

### Patents

This study is currently filed for a provisional patent.

## Supplementary Information

ESM 1 (CSV 9223 kb)

ESM 2 (CSV 2606 kb)
